# Impact of family-centered care combined with the transtheoretical model on health behaviors of children with nephrotic syndrome

**DOI:** 10.12669/pjms.42.4.15552

**Published:** 2026-04

**Authors:** Jingxia Wang, Huan Zhang, Yanjun Yang, Xiaohong Xi, Licai Gu

**Affiliations:** 1 Jingxia Wang, Department of Nephrology and Immunology, Hebei Children’s Hospital, Hebei Provincial Clinical Medical Research Center for Children’s Health and Diseases, Shijiazhuang, Hebei Province 050030, P.R. China; 2 Huan Zhang, Department of Nephrology and Immunology, Hebei Children’s Hospital, Hebei Provincial Clinical Medical Research Center for Children’s Health and Diseases, Shijiazhuang, Hebei Province 050030, P.R. China; 3 Yanjun Yang, Department of Nephrology and Immunology, Hebei Children’s Hospital, Hebei Provincial Clinical Medical Research Center for Children’s Health and Diseases, Shijiazhuang, Hebei Province 050030, P.R. China; 4 Xiaohong Xi, Department of Nephrology and Immunology, Hebei Children’s Hospital, Hebei Provincial Clinical Medical Research Center for Children’s Health and Diseases, Shijiazhuang, Hebei Province 050030, P.R. China; 5 Licai Gu Department of Pediatrics, the First Hospital of Hebei Medical University, Shijiazhuang, Hebei Province 050030, P.R. China

**Keywords:** Family-centered Care, Health Behaviors, Nephrotic Syndrome, Transtheoretical Model

## Abstract

**Objective:**

To evaluate the potential impact of family-centered care combined with the transtheoretical model on the health behaviors of children with nephrotic syndrome.

**Methodology:**

A retrospective analysis was conducted on the clinical data of 150 children with nephrotic syndrome admitted to the Department of Nephrology and Immunology of Hebei Children’s Hospital from June 2022 to June 2025. Based on the different nursing intervention protocols, the children were divided into two groups: 73 children who received routine nursing care were assigned to the control group, and 77 children who received family-centered care combined with the transtheoretical model intervention were assigned to the study group. The health behavior compliance rates, clinical outcome measures, complications, and family members’ nursing satisfaction were statistically compared between the two groups.

**Results:**

The health behavior compliance rate of the study group (94.81%) was significantly higher than that of the control group (83.56%) (P<0.05). After intervention, the 24-hour urinary protein levels and total cholesterol levels decreased in both groups compared with before intervention, while serum albumin levels increased, with the study group showing better outcomes than the control group (P<0.05). The incidence of complications in the study group (6.49%) was lower than that in the control group (17.81%) (P<0.05). The nursing satisfaction of the children’s family members in the study group (94.81%) was higher than that in the control group (82.19%) (P<0.05).

**Conclusion:**

The implementation of family-centered care, combined with the transtheoretical model, in the nursing of children with nephrotic syndrome was associated with significant improvements in health behaviors and compliance rates, potentially optimizing disease control and contributing to a lower risk of complications, and enhancing the satisfaction of children’s family members with nursing.

## INTRODUCTION

Nephrotic syndrome is a common chronic immune-mediated pediatric disease of the urinary system. It is characterized by massive proteinuria, hypoproteinemia, edema, and hyperlipidemia, and is associated with a protracted and recurrent course, and long treatment cycle, which requires long-term adherence to strict dietary control, medication compliance, and lifestyle interventions.^1^,^2^ Unhealthy behaviors (such as arbitrary withdrawal of medication, uncontrolled diet, and lack of moderate exercise) can not only lead to recurrent and aggravated conditions, increase the risk of complications such as steroid dependence and infection, but also affect the growth and development as well as mental health of children, imposing a heavy burden on families.^3^,^4^

Traditional disease-oriented nursing models for pediatric nephrotic syndrome often relegate family members to passive bystanders, failing to address the critical gaps in family empowerment and organizational support.^5^ Furthermore, prescriptive health education typically ignores the patient’s dynamic stage of behavioral readiness, leading to poor long-term adherence.^6^ Family-centered care (FCC) fills these gaps by restructuring the clinical-family partnership into a supportive care environment,^7^,^8^ while the Transtheoretical Model (TTM) provides a scientific behavioral driving mechanism by tailoring interventions to specific stages of change.^9^ This synergy creates a closed-loop system of ’environmental support plus mechanistic drive,’ addressing both the context and the process of health behavior modification. In contrast, family-centered care emphasizes the child’s family as the core care unit. This approach meets the physical, psychological, and social needs of children through collaboration between medical staff and families, demonstrating significant advantages in the nursing care of children with chronic diseases.^7^ The transtheoretical model focuses on the dynamic process of individual behavior change, dividing it into five stages: precontemplation, contemplation, preparation, action, and maintenance. Through targeted, staged intervention strategies, the transtheoretical model stimulates motivation for individual behavior change and improves the precision and effectiveness of interventions.^10^

This retrospective study combined family-centered care with the transtheoretical model to construct a staged, family-participatory health behavior intervention program. The objective was to clarify the current status of health behaviors among children with nephrotic syndrome and the level of caregiving ability of their families, analyze the distribution characteristics of behavioral change stages in children, explore the improvement effect of this combined intervention program on core health behaviors of children, and simultaneously evaluate its impact on disease control, complication rate, and satisfaction of family caregivers. Ultimately, this aimed to provide scientific evidence-based and scalable practical solutions for optimizing health behavior intervention strategies and enhancing nursing quality for children with nephrotic syndrome in clinical settings.

## METHODOLOGY

Clinical data of 150 children with nephrotic syndrome admitted to the Department of Nephrology and Immunology of Hebei Children’s Hospital from June 2022 to June 2025 were retrospectively analyzed. Participants were assigned to either the study group (n=77) or the control group (n=73) based on the specific clinical nursing pathways implemented in their respective wards during the study period. Both groups were enrolled within the same time window (June 2022 to June 2025) and were managed by the same multidisciplinary medical team in the Department of Nephrology and Immunology to ensure consistency in basic clinical care. This ward-based allocation was determined by institutional protocols rather than family preference or individual motivation, effectively minimizing potential selection bias. Furthermore, to prevent inter-group contamination, the FCC+TTM integrated care protocol and routine care were executed independently in separate wards by nursing staff who strictly adhered to their assigned standardized protocols.

### Ethical approval:

This retrospective study was approved by the Ethics Committee of Hebei Children’s Hospital (No. 202136; May 17, 2021), which granted a waiver of informed consent due to the minimal risk involved. To ensure data security, all personal identifiers were de-identified using randomized codes. Online follow-ups via professional departmental WeChat accounts utilized standardized scripts and strictly prohibited the sharing of sensitive medical images in group chats to maintain patient confidentiality.

### Inclusion criteria:

- Conforming to the diagnostic criteria of nephrotic syndrome,^11^ confirmed by routine urine test, 24-hour urinary protein quantitative test and blood biochemical examination.
- Aged 3–14 years old with a course of disease ≥ 3 months.
- Receiving hormone therapy or hormone combined with immunosuppressant therapy.
- Receiving long-term care from fixed family caregivers (parents or main guardians).

### Exclusion criteria:

- Complicated with secondary nephrotic syndrome such as congenital kidney disease, lupus nephritis and purpura nephritis.
- Complicated with vital organ insufficiency.
- In the acute stage of infectious diseases.
- With mental retardation, attention deficit hyperactivity disorder, etc.
- Having severe complications such as acute kidney injury, severe infection and thromboembolism during the course of disease.

### Routine nursing care approach:

The routine nursing care included:

### Disease knowledge education:

After admission, a centralized health lecture was held once for the children and their family members, and disease nursing manuals were distributed to explain the treatment principles of nephrotic syndrome, precautions for hormone medication, dietary taboos (low-salt, low-fat, high-quality protein), and key points for the prevention of complications such as edema and infection.

### Daily nursing guidance:

During hospitalization, daily condition monitoring (urine output, urine color, edema degree, vital signs) was carried out. Family members were instructed to complete the children’s skin care, oral cleaning, and activity management (avoid strenuous exercise and prevent trauma). Written discharge guidance was provided upon discharge, specifying the review time and medication dosage.

### Basic follow-up communication:

Telephone follow-ups were conducted at the 1st and 3rd months after discharge to inquire about the children’s medication status, diet, exercise, and disease changes, and to answer simple inquiries from family members. No personalized intervention was implemented. *Psychological support*. When anxiety was observed in children or their family members, verbal comfort was given to alleviate psychological pressure.

### Family-centered care combined with the transtheoretical model intervention:

The approach was implemented by combining the five stages of behavior change in the transtheoretical model (precontemplation, contemplation, preparation, action, and maintenance) as the framework, with the collaborative care concept of family-centered care. To ensure reproducibility and provide comprehensive operational details, a structured description of the FCC+TTM intervention program—developed in accordance with the TIDieR (Template for Intervention Description and Replication) checklist—is provided in Supplementary Table-I. This includes the mapping of TTM stages to specific behavior change techniques (BCTs), standardized follow-up scripts, and incentive criteria. Key tool samples, such as the stage assessment form and the family education package outline, are included in Appendix-I(A-C). A staged and family-participatory intervention program was formulated, as detailed below:

**Supplementary Table-I:** Structured description of the Family-Centered Care combined with the Transtheoretical Model (FCC+TTM) intervention based on the TIDieR checklist.

TIDieR Item	Description
1. Brief Name	Family-Centered Care Combined with the Transtheoretical Model (FCC+TTM) Intervention.
2. Why (Rationale)	To address the gaps in traditional “disease-oriented” models by empowering the family as the core care unit (FCC) and aligning educational strategies with the patient’s and family’s readiness for behavior change (TTM). This dual-core approach aims to improve health behaviors, optimize disease control, and reduce complications in pediatric nephrotic syndrome.
3. What (Materials)	1. Assessment Tools: Transtheoretical Model Stage Assessment Scale, Quantitative Assessment Scale for Health Behaviors of Children with Nephrotic Syndrome.
	2. Educational Materials: Customized nursing guidance manuals, age-appropriate diet/exercise programs.
	3. Support Tools: Medication reminder apps, diet record forms, health behavior point-reward system.
4. What (Procedures & BCTs Mapping)	Assessment Stage (Week 1): Assess baseline behavior and family capacity using semi-structured interviews and scales .
	Precontemplation (BCT: Information about health consequences): Conduct one-on-one communication, analyze the harms of unhealthy behaviors, and invite families with well-controlled cases to share experiences to stimulate motivation .
	Contemplation (BCT: Goal setting & Action planning): Set personalized, actionable small-step health behavior goals (e.g., daily low-salt diet) and provide customized manuals .
	Preparation (BCT: Instruction on how to perform the behavior): Organize practical training on edema measurement, qualitative urine protein testing, and correct hormone administration .
	Action (BCT: Behavioral practice & Reward): Monitor implementation via weekly follow-ups, correct deviations, and provide positive incentives (redeeming small gifts with health points) .
	Maintenance (BCT: Relapse prevention & Social support): Establish long-term consultation channels, conduct bi-weekly follow-ups, and summarize caregiving experiences to form sustainable habits.
5. Who Provided	A multidisciplinary intervention team consisting of pediatricians, specialist nurses, dietitians, and psychotherapists.
6. How (Mode of Delivery)	Face-to-face (centralized lectures, one-on-one communication, practical training, home visits) and online communication (weekly/bi-weekly WeChat or telephone follow-ups, online group exchanges).
7. Where (Location)	Department of Nephrology and Immunology at Hebei Children’s Hospital, patients’ homes, and secured online platforms (WeChat).
8. When and How Much (Dosage)	Total observation period: 3 months. Frequency: Weekly follow-ups during the Action stage; Monthly home visits or online group exchanges; Bi-weekly follow-ups during the Maintenance stage.
9. Tailoring	Interventions were highly personalized. Dietitians adjusted diet plans monthly based on blood biochemical indicators. Psychotherapists customized cognitive behavioral or play therapies for children experiencing low self-esteem. Intervention methods were also adapted to the family’s economic conditions and cultural background.
10. Modifications	N/A (Retrospective study; the standardized protocol was consistently applied throughout the designated period).
11. How Well (Planned Fidelity)	Fidelity was monitored by the multidisciplinary team during weekly/bi-weekly follow-ups and evaluated using the self-designed Quantitative Assessment Scale for Health Behaviors (scoring range 0-40).
12. How Well (Actual Fidelity)	High intervention fidelity and adherence were achieved, with the study group demonstrating a 94.81% health behavior compliance rate (66.23% full compliance, 28.57% basic compliance).

### Assessment Stage (Week 1 of Intervention):

An intervention team consisting of pediatricians, specialist nurses, dietitians, and psychotherapists was established. Through semi-structured interviews, questionnaires, and medical record reviews, the current status of the children’s health behaviors (diet, medication adherence, exercise, and self-monitoring) and the family’s caregiving capacity were assessed. The Transtheoretical Model Stage Assessment Scale was used to determine the behavior change stages of the children and their family members, providing a basis for personalized interventions.

### Implementation of staged interventions:

### Precontemplation stage (No Intention to Change):

One-on-one in-depth communication was conducted with family members. Combined with cases of recurrent illness in children, the harms of unhealthy behaviors were analyzed to strengthen their awareness of the correlation between disease treatment and healthy behaviors. Families of children with well-controlled conditions were invited to share their caregiving experiences, which stimulated the participating families’ motivation to change and helped them establish the belief that healthy behaviors can improve prognosis.

### Contemplation stage (Intention to Change but No Plan):

The intervention team worked with family members to set personalized health behavior goals (e.g., “adhere to a low-salt diet daily” and “take medication on time without arbitrary dosage reduction”), which were broken down into actionable small steps. Customized nursing guidance manuals were provided, and diet plans and exercise programs tailored to the children’s ages (e.g., mild indoor exercise routines for school-age children) were designed, clarifying the family members’ caregiving responsibilities and implementation standards.

### Preparation stage (Having a Plan and Preparing to Act):

The team assisted families in building a caregiving support system, such as guiding family members to use medication reminder apps and diet record forms. Practical training sessions were organized for family members, covering edema measurement, qualitative urine protein testing methods, and the correct administration of hormone drugs. The schedules of family caregivers were coordinated to ensure the consistent implementation of intervention measures.

### Action stage:

Follow-up was conducted once a week via WeChat or telephone to monitor the implementation of health behaviors, and timely corrections were made for deviations (e.g., excessive dietary salt intake and inappropriate exercise intensity). A home visit or online group exchange was held monthly to address practical problems encountered during caregiving (e.g., children’s resistance to special diets and missed medication doses at school). Positive incentives (e.g., redeeming small gifts with health behavior points) were provided to families with active compliance to strengthen behavior persistence.

### Maintenance stage:

Follow-up was conducted every two weeks to assess maintenance of healthy behaviors, and intervention strategies were adjusted accordingly (e.g., optimizing diet plans based on the stability of the children’s conditions). Family members and children were guided to jointly summarize caregiving experiences, forming a long-term, sustainable, healthy behavior model. A long-term consultation channel was established to provide follow-up care for families and prevent relapse of behavior.

### Strengthening family collaborative care:

Throughout the intervention, family members were regarded as core caregiving partners. Regular family seminars were organized for them to share caregiving experiences and challenges. Psychological counseling services were provided to alleviate the anxiety and psychological stress of family caregivers. Intervention methods were adjusted to the family’s economic conditions and cultural background to ensure feasibility and acceptability.

### Multidisciplinary collaborative support:

Dietitians adjusted the children’s diet plans monthly based on their blood biochemical indicators (e.g., albumin and blood lipid levels). Psychotherapists implemented play therapy or cognitive behavioral interventions for children who might experience low self-esteem or resistance. Pediatricians regularly assessed the children’s conditions and optimized treatment plans, forming a multidimensional, collaborative intervention approach.

### Observation indicators:

- Health behaviors were assessed using a self-designed Quantitative Assessment Scale for Health Behaviors of Children with Nephrotic Syndrome (Cronbach’s α = 0.89, content validity index = 0.92). The scale covers 4 dimensions with 20 items: diet management (low-salt and low-fat intake, high-quality protein matching, avoidance of taboo foods, etc.), medication adherence (timely medication taking, dosage compliance, no arbitrary withdrawal/ dosage modification), exercise and daily routine (moderate activity, avoidance of overfatigue, regular work and rest), and self-monitoring (urine output observation, edema identification, reporting of discomfort). Each item was scored on a 3-point scale (0 = not performed, 1 = partially performed, 2 = fully performed), yielding a total score of 0 to 40. A score ≥ 35 indicated full compliance, 28–34 indicated basic compliance, and < 28 indicated non-compliance. The health behavior compliance rate was calculated as (number of cases with full compliance + number of cases with basic compliance) divided by the total number of cases, multiplied by 100%. Assessments were conducted once before the intervention and once three months after.
- Clinical outcome measures, including 24-hour urinary protein quantification, serum albumin, and total cholesterol. Venous blood and urine samples were collected before and 3 months after the intervention and analyzed using an automatic biochemical analyzer.
- Complications were identified through the hospital’s Electronic Medical Record (EMR) system and standardized clinical assessments during the 3-month follow-up. Standardized diagnostic criteria were applied:
- Infection was confirmed by clinical symptoms (e.g., fever, cough, or skin lesions) and laboratory evidence (elevated WBC count, positive C-reactive protein, or positive culture);
- Electrolyte disturbances were defined by biochemical thresholds of serum sodium < 135 mmol/L (hyponatremia) or serum potassium < 3.5 mmol/L (hypokalemia);
- Cushing’s syndrome was identified by typical physical signs of exogenous steroid use (e.g., moon face, buffalo hump). All recorded complications were clinically significant events requiring intervention, such as intravenous therapy, brief hospitalization, or tailored adjustments to steroid regimens.
- Nursing satisfaction of children’s family members, evaluated using a self-designed Likert 5-point scale (Cronbach’s α = 0.91, content validity index = 0.93). The scale includes 25 items across four dimensions: intervention individualization, guidance practicality, communication timeliness, and support comprehensiveness, with a total score of 100. A score ≥ 90 indicated very satisfied, 75–89 indicated satisfied, 60–74 indicated general, and < 60 indicated dissatisfied. The total satisfaction rate was calculated as (number of cases with very satisfied + number of cases with satisfied) divided by the total number of cases × 100%.

### Statistical analysis:

Data was analyzed using SPSS 25.0. Measurement data were described as (*x̅*+*s*) and tested by the t-test. Count data were described as frequencies and constituent ratios (%) and tested by the *χ^2^*-test. A P value < 0.05 indicated a statistically significant difference.

## RESULTS

There were no statistically significant differences in baseline data such as gender, age, course of disease, type of onset, pathological type, hormone treatment stage, and relevant data of family caregivers between the two groups (P>0.05), indicating good comparability (Table-I). As shown in Table-II, the health behavior compliance rate of the study group (94.81%) was higher than that of the control group (83.56%) (P < 0.05).

**Table-I:** Comparison of general data between the two groups.

Items	Study Group (n=77)	Control Group (n=73)	t/χ^2^	P
Age(*x̅*+*s*, years)	7.59±2.30	7.75±2.42	0.415	0.679
***Gender[n(%)]***				
Male	45(58.44)	40(54.79)	0.203	0.652
Female	32(41.56)	33(45.21)		
Course of Disease(*x̅*+*s*, years)	1.90±0.75	1.86±0.82	0.312	0.756
Type of Onset[n(%)]				
Initial Onset	51(66.23)	46(63.01)	0.170	0.680
Recurrence	26(33.77)	27(36.99)		
***Stage of Hormone Therapy[n(%)]***				
Induction Remission Stage	29(37.66)	30(41.10)	0.185	0.667
Consolidation and Maintenance Stage	48(62.34)	43(58.90)		
Family Caregivers[n(%)]				
Parents	70(90.91)	69(94.52)	0.719	0.396
Others	7(9.09)	4(5.48)		
***Educational Level of Family Caregivers[n(%)]***				
College and Above	41(53.25)	38(52.05)	0.031	0.985
Senior High School	22(28.57)	21(28.77)		
Junior High School and Below	14(18.18)	14(19.18)		

**Table-II:** Comparison of health behaviors between the two groups [n (%)].

Group	Number of Cases	Full Compliance	Basic Compliance	Non-Compliance	Compliance Rate
Study Group	77	51(66.23)	22(28.57)	4(5.19)	73(94.81)
Control Group	73	38(52.05)	23(31.51)	12(16.44)	61(83.56)
*χ^2^*					4.972
*P*					0.026

There were no significant differences in 24-hour urinary protein excretion, serum albumin, and total cholesterol between the two groups before intervention (P>0.05). After intervention, the levels of 24 hours urinary protein quantification and total cholesterol in both groups decreased compared with those before intervention, while the serum albumin levels increased, with the study group showing better results than the control group (P<0.05) (Table-III).

**Table-III:** Comparison of disease control effects between the two groups(*x̅*+*s*).

Time	Group	Number of Cases	24-hour Urinary Protein Quantification (g/24h)	Serum Albumin (g/L)	Total Cholesterol (mmol/L)
Before Intervention	Study Group	77	4.89±1.72	17.45±5.32	12.77±3.56
	Control Group	73	5.05±1.59	18.07±4.98	13.01±3.24
	*t*		0.591	0.736	0.431
	*P*		0.556	0.463	0.667
After Intervention	Study Group	77	0.10±0.03	49.45±4.67	3.64±1.51
	Control Group	73	0.17±0.06	44.82±5.85	4.71±1.66
	*t*		9.108	5.371	4.133
	*P*		<0.001	<0.001	<0.001

The incidence of complications in the study group (6.49%) was considerably lower than that in the control group (17.81%) (P<0.05) (Table-IV and Table-V, shows that the nursing satisfaction rate of the family members in the study group (94.81%) was significantly higher than in the control group (82.19%) (P<0.05).

**Table-IV:** Comparison of complications between the two groups(%)].

Group	Number of Cases	Respiratory Tract Infection	Skin Infection	Electrolyte Disturbance	Cushing’s Syndrome	Total Incidence Rate
Study Group	77	2(2.60)	1(1.30)	1(1.30)	1(1.30)	5(6.49)
Control Group	73	4(5.48)	2(2.74)	4(5.48)	3(4.11)	13(17.81)
*χ^2^*						4.543
*P*						0.033

**Table-V:** Comparison of nursing satisfaction of family members of children between the two groups[n(%)].

Group	Number of Cases	Very Satisfied	Satisfied	General	Dissatisfied	Total Satisfaction Rate
Study Group	77	45(58.44)	28(36.36)	4(5.19)	0(0.00)	73(94.81)
Control Group	73	36(49.32)	24(32.88)	9(12.33)	4(5.48)	60(82.19)
*χ^2^*						5.933
*P*						0.015

## DISCUSSION

This study demonstrates that family-centered care (FCC) combined with the transtheoretical model (TTM) significantly enhances health behaviors, clinical outcomes, and family satisfaction in pediatric nephrotic syndrome (NS).^1^,^5^,^6^ Traditional routine nursing often fails due to one-way communication and a lack of family empowerment, leading to poor adherence, frequent recurrence, and substantial caregiver burden.^10^,^12^ In contrast, the synergistic FCC+TTM framework addresses these gaps: the FCC component establishes a collaborative care environment to bolster caregiver capacity,^5^,^8^,^11^ while the TTM provides staged navigation to align interventions with behavioral readiness.^6^,^9^ This precise synchronization ensures that management—such as dietary control and medication adherence—is perfectly matched to the child’s behavioral stage, thereby minimizing relapse risks and stabilizing laboratory markers.^13^-^15^

The study group achieved a significantly higher compliance rate (94.81%) than the control group (83.56%).^13^,^16^ This confirms that family-centric models standardize health behaviors by reinforcing caregiver responsibilities and capabilities.^5^,^11^ Staged TTM interventions further improve precision by driving transitions through cognitive and behavioral change processes.^6^,^12^ While pure FCC models may have limited impact on long-term behavior maintenance,^7^,^8^ the integration of staged guidance effectively stimulates motivation and consolidates habits through a closed-loop process of planning and reinforcement.^9^,^10^,^16^

Superior improvements in 24-hour urinary protein, serum albumin, and total cholesterol levels in the study group were directly associated with increased compliance.^1^,^11^,^16^ Standardized health management, including rigorous home monitoring and medication reminders, is essential to reduce recurrence in steroid-sensitive NS.^11^,^15^ These findings parallel results by Yao L et al.^16^ and Wang H et al.^13^, illustrating that TTM-based interventions optimize disease control through sustained treatment adherence and physiological stabilization.^13^,^14^

Furthermore, the study group showed a lower incidence of complications (6.49%) compared to the control group (17.81%).^3^,^4^ This reduction is attributed to standardized care practices, such as skin and dietary hygiene, which mitigate infection risks—the most common complication in NS.^6^,^14^ TTM also improved adherence to hormone protocols, thereby reducing the incidence of aggravated side effects like Cushing’s syndrome.^9^,^14^ Enhanced family satisfaction (94.81%) reflects the benefits of improved clinical-family communication and increased caregiver confidence in managing the condition.^8^,^11^

### Strengths of the study:

Key strengths of this research include the innovative fusion of family support with scientific behavior change mechanisms and a multidisciplinary approach addressing both biochemical and psychosocial health.^5^,^6^,^13^ To build on these results, future research should utilize extended follow-up periods (6–12 months) to evaluate long-term behavior sustainability.^10^ Subsequent studies must incorporate harder clinical endpoints, such as relapse frequency, cumulative steroid dosage, and healthcare utilization rates, within large-scale multicenter designs to validate the generalizability of this combined model.^11^,^12^

### Limitations:

Several limitations warrant consideration. First, as a single-center retrospective study, this research provides Level III evidence and may be subject to inherent selection bias. Despite comparable baseline hormone therapy stages (P=0.667), the absence of granular daily dosage and tapering data prevents excluding potential pharmacotherapeutic confounding on biochemical outcomes; thus, these findings represent preliminary associations rather than definitive causal links. Second, the limited sample size (n=150) precluded robust exploratory subgroup or interaction analyses, as further stratification would have compromised statistical power and increased the risk of Type-I errors. Finally, the three-month follow-up primarily captures the “action” stage of behavioral change but is insufficient to evaluate long-term maintenance or chronic outcomes such as relapse frequency and healthcare utilization. Future 6–12 month prospective research incorporating quality of life (QoL) scales and harder clinical endpoints is essential to definitively assess the long-term sustainability and impact of the FCC+TTM intervention.

**Appendix-IA:** Transtheoretical Model (TTM) Stage Assessment Tool (Sample).

***Purpose:*** To determine the behavior change stage of the family caregiver and the child regarding core nephrotic syndrome (NS) management (e.g., diet control, medication adherence).
***Instructions:*** Please select the statement that best describes your current attitude and behavior towards strictly following the NS health management plan (including a low-salt diet, strict medication compliance, and daily urine/edema monitoring):
• [ ] Precontemplation: We do not currently follow the strict health management plan, and we do not intend to start within the next 6 months. (We believe it is too difficult or unnecessary).
• [ ] Contemplation: We are not currently following the strict plan, but we are seriously thinking about starting it within the next 6 months.
• [ ] Preparation: We have decided to strictly follow the plan and are taking steps to prepare (e.g., buying low-sodium salt, downloading a medication reminder app), aiming to start within the next 30 days.
• [ ] Action: We have been strictly following the health management plan, but for less than 6 months. It still requires conscious effort.
• [ ] Maintenance: We have been strictly following the health management plan for more than 6 months. It has become a stable daily habit.

## CONCLUSION

The implementation of family-centered care, combined with the transtheoretical model, in the intervention for children with nephrotic syndrome was associated with significant improvements in the compliance rate with health behaviors, may contribute to better disease control, and is linked to a reduced risk of complications, and enhancing the satisfaction of children’s family members with nursing. This combined intervention model addresses the limitations of traditional nursing, provides a scientifically grounded and feasible program for long-term nursing care of children with nephrotic syndrome, and has high clinical value.

**Appendix-IB:** Standardized WeChat Follow-up Scripts.

***Purpose:*** To ensure consistent and structured communication during the Action and Maintenance stages, minimizing information bias.
***1. Action Stage (Weekly Follow-up Script):***
“Hello [Caregiver's Name], this is the pediatric nephrology nursing team from Hebei Children's Hospital. It's time for our weekly check-in.
***1. Medication Check:*** Has [Child's Name] taken their prescribed steroid/immunosuppressant medication at the exact dosages this week? Were there any missed doses?
***2. Diet & Edema Check:*** Have you been able to maintain the low-salt and low-fat diet? Have you noticed any new swelling around the eyes or lower limbs?
***3. Urine Monitoring:*** What were the results of the qualitative urine protein tests this week?
***4. Troubleshooting:*** Did you encounter any difficulties managing the child's diet or medication this week? How can we assist you?
***Goal for next week:*** Let's continue to track the daily urine output and ensure no outside snacks high in sodium are consumed.”
***2. Maintenance Stage (Bi-weekly Follow-up Script):***
“Hello [Caregiver's Name], great job maintaining the routine! This is our bi-weekly check-in.
***1. Stability Check:*** Has the daily routine, medication, and diet remained stable over the past two weeks?
***2. Relapse Prevention:*** Have there been any signs of respiratory infections or fever? (Remind them of infection prevention protocols).
***3. Psychological Support:*** How is [Child's Name] feeling about school and daily activities? Are you feeling overwhelmed?
Remember, our long-term consultation channel is always open if you need immediate advice.”

**Appendix-IC:** Health Behavior Incentive Criteria (Point-Reward System).

Target Behavior	Point Allocation	Verification Method
Medication Adherence	2 Points / Week	Uploading weekly medication check-off log via WeChat.
Dietary Management	2 Points / Week	Submitting 3 random daily meal photos meeting low-salt/low-fat criteria.
Self-Monitoring	2 Points / Week	Recording daily weight, edema status, and qualitative urine protein.
Active Participation	5 Points / Session	Attending the monthly online group exchange or family seminar.
Reward Tiers:	**20 Points:** NS-friendly nutritional recipe booklet.	
	**40 Points:** Customized electronic medication reminder pillbox.	
	**80 Points:** Age-appropriate educational toys/books for the child.	

***Purpose:*** To provide positive reinforcement for families in the Action stage to strengthen behavior persistence.

### Authors’ contributions:

**JW and HZ:** Literature search, study design and manuscript writing.

**YY, XX and LG**: Data collection, data analysis and interpretation. Critical Review.

**JW and HZ:** Manuscript revision and validation and is responsible for the integrity of the study.

All authors have read and approved the final manuscript.
